# Next-Generation Myopia Control Spectacles Hint at Defocus Opponency Circuits in the Retina

**DOI:** 10.1007/s44402-026-00129-w

**Published:** 2026-06-17

**Authors:** Jeremy A. Guggenheim, John R. Phillips

**Affiliations:** 1 School of Optometry & Vision Sciences, Cardiff University, Cardiff, UK; 2 School of Optometry and Vision Science, The University of Auckland, Auckland, New Zealand

Myopia management is becoming a routine part of primary eye care, informed by evidence from high-quality randomised controlled trials [1, 2]. The first optical treatments for myopia were bifocal and progressive addition spectacles, designed to reduce retinal blur from accommodative lag. Later, ‘dual focus’ myopia control contact lenses with concentric zones of alternating power were introduced; these lenses corrected the patient’s distance refractive error while also providing additional plus power that focused some of the incident light in front of the retina in peripheral regions [3, 4]. The peripheral myopic defocus produced by dual focus contact lenses was designed to generate a ‘STOP’ signal in the retina; animal studies suggest that the defocus experienced by a myopic eye generates an equivalent ‘STOP’ signal to temporarily inhibit further eye growth [5]. Interestingly, in animal models, fitting a plus lens in front of one or both eyes inhibits further growth in the treated eye or eyes. However, in children, bilateral under-correction of myopia is not effective, while monocular under-correction of the non-dominant eye is effective in slowing myopia progression in that eye [6, 7]. In the monocular situation, the non-dominant eye experiences myopic defocus during both near and distance viewing, whereas in bilateral under-correction, both eyes obtain sharp vision when viewing at near. In animal studies, brief periods of sharp vision can counteract prolonged periods of myopic defocus [8]; this may explain why bilateral under-correction in children is ineffective.

Even before the introduction of dual-focus contact lenses, orthokeratology (ortho-k) lenses had been shown to slow myopia progression, but the underlying mechanism was unclear [9]. Subsequently, researchers realised that the corneal flattening profile in eyes fitted with ortho-k lenses generated a ring of plus power that also introduced peripheral myopic defocus; this suggested that ortho-k and dual-focus myopia control contact lenses slow myopia progression via the same mechanism [10]. More recently, myopia control spectacle lenses have been developed that possess an array of discrete, positively-powered lenslets—small, circular segments about 1 mm in diameter—that produce additional plus power. Lenslet spectacles have a clear central zone approximately 10 mm in diameter that provides a focused image at the fovea when the patient is looking straight ahead at a distant target. The midperiphery or remainder of the lens contains hundreds of lenslets in a honeycomb pattern or concentric ring arrangement. Each lenslet produces a non-coaxial focus in front of the retina such that the carrier lens and lenslets provide adjacent and overlapping regions of ‘competing defocus’ across the midperipheral retina [11]. As with dual-focus contact lenses, the underlying theory for the myopia control effect of lenslet spectacles is midperipheral myopic defocus that generates a retinal ‘STOP’ signal [12, 13].

A still more recent type of myopia management spectacle lens, Diffusion Optics Technology (DOT^®^; sightglassvision.com) lenses, have a uniform refractive power that corrects the patient’s distance vision; these lenses have a clear central zone while the remaining regions contain tiny light scattering elements. An eye viewing a distant target straight ahead through a DOT lens will receive a focused image at the fovea, while the midperipheral retina receives a lower contrast image in which high spatial frequency content is lost. The underlying theory for the myopia control effects of DOT lenses is based on the concept that the retina interprets mild contrast reduction as a cue that the eye is too long, thus generating a ‘STOP’ signal [14].

Key unresolved questions about optical myopia control interventions include the following. Firstly, form deprivation—strongly scattering the visual image so that both high and low spatial frequency information is lost—produces a strong ‘GO’ signal that promotes eye growth and myopia [15, 16]. Why, then, does the mild scattering of the visual image by DOT lenses slow myopia progression rather than accelerating it? Secondly, spectacles with negatively powered lenslets slow children’s myopia progression to a similar extent as spectacles with positively powered lenslets [17]. Does this mean that lenslets exert their effects by contrast reduction, similar to DOT lenses, rather than by producing myopic defocus [18, 19]?

Coming to the present day, preliminary reports suggest the performance of second-generation lenslet spectacles for myopia control exceeds that of the original designs [20, 21]. Although the long-term efficacy of these second-generation lenses has not yet been reported in peer-reviewed journal articles, the preliminary findings suggest that by increasing the positive power of the lenslets, the myopia control efficacy of lenslet spectacles has been improved. As discussed below, if confirmed, these results offer intriguing insights into the underlying mechanism of myopia control.

When lens manufacturers first thought about increasing the power of lenslets in second-generation lenses, they would have been aware of the theoretical risk of inducing form deprivation, since primate studies have shown that myopic defocus beyond a threshold level produces a ‘GO’ signal (or no signal at all) rather than a ‘STOP’ signal [22]. Specifically, monkeys have been found to emmetropise accurately when reared wearing −3.00 or +3.00 D single vision lenses, but poorly when wearing −6.00 or +6.00 D lenses [22]. We speculate that the areas of sharp focus adjacent to and overlapping with areas of low contrast blur may be critical to the mechanism of action of ortho-k, dual-focus contact lenses, lenslet lenses and DOT lenses. In other words, perhaps simultaneous exposure to both sharp and blurred images is critical to the eye, directing its growth signal towards ‘STOP’ instead of ‘GO’. Thus, whereas exposure to severe blur from a +5.00 or + 6.00 D lens may be sufficient to induce form deprivation myopia when experienced uniformly across the retina, the same magnitude of blur might generate a strong ‘STOP’ signal when detected alongside areas of sharp focus. Moreover, because the direction of gaze can move relative to the lenslets on a spectacle lens, lenslet spectacles can create temporally and spatially varying patterns of defocus across the retina, whereas contact lenses are limited to spatially varying patterns. In theory, this may provide spectacle-based treatments with greater potential treatment efficacy than contact lens-based treatments. With regard to DOT lenses, perhaps the existence of the central clear zone is critical in producing areas of simultaneous sharp and blurred image, sufficient to ensure a ‘STOP’ signal is generated, rather than the ‘GO’ signal expected if contrast was reduced uniformly across the whole retina, as in form deprivation.

The retina relies heavily on opposing neural signals to detect contrast (centre-surround receptive fields) and colour (colour opponency) [23, 24]. If, as we hypothesise, the retina is able to simultaneously detect sharp and blurred images and integrate this information to determine the sign-of-defocus, then this brings to mind the idea of a retinal ‘defocus opponency’ mechanism. While we are not aware of physiological evidence to support such a circuit, interpreting the results of clinical trials of interventions for controlling myopia progression in the context of defocus opponency may have value. For example, if there is no risk of excessive blur from lenslets inducing form deprivation, then perhaps maximally effective myopia control spectacles could be manufactured using lenses with lenslets of very high plus power or lenslets that scatter light at all spatial frequencies; preliminary results testing diffuser lenslets support this idea [25]. To finish, we note that defocus opponency fails to explain a previous finding reported by Park et al. [26]; these authors found that chicks raised wearing a diffuser developed form deprivation myopia, while chicks wearing a diffuser on top of a plus lens developed hyperopia. Defocus opponency cannot explain this result since all regions of the retina of the chicks wearing a diffuser on top of a plus lens received the same stimulus. Therefore, additional cues to the sign-of-defocus, such as chromatic cues, are also required to explain existing research findings [27].

## Data Availability

No datasets were generated or analysed during the current study.
